# New horizons in the reproductive biology of Chinese pangolin (*Manis pentadactyla*) using the gonadal hormonal profile

**DOI:** 10.1038/s41598-023-43237-0

**Published:** 2023-10-03

**Authors:** Bharti Arora, Kurtis Jai-Chyi Pei, Shih-Chien Chin

**Affiliations:** 1 Department of Natural Resources and Environmental Science, National Dong Hwa University, Hualien, 974301 Taiwan; 2 Institute of Wildlife Conservation, College of Veterinary Medicine, National Pingtung University of Science and Technology, Pingtung, 91201 Taiwan; 3 Taiwan Wildlife Society, 900 Pingtung, Taiwan; 4 Taipei Zoo, Taipei, 11656 Taiwan; 5 Chin’s Animal Hospital, Taipei, 11656 Taiwan

**Keywords:** Biological techniques, Physiology, Zoology, Endocrinology

## Abstract

The reproductive uniqueness of pangolins has been documented through diverse biological reports with discernible data discrepancies in gestation, copulation, and pregnancy. These mechanistic reproductive differences have yet to be endocrinologically quantified, which could assist in optimizing natural breeding in zoos to recover endangered species. The present research characterizes the Chinese pangolin's annual seasonal reproductive pattern by measuring immunoreactive estrogens and progestagens in 34 captive females and testosterone in 29 captive males. Our results showed that Chinese pangolins are seasonal breeders, with most births witnessed during Sept–Dec, overlapping with the field records. Females exhibited spontaneous ovulation and post-partum ovulation. Pregnant females exhibited a higher P4 level for ~ 9 months (Jan–Sept) and decreased before parturition (Oct–Dec). The circulating E2 is maintained at the baseline in pregnant females year-round. Contrastingly, in non-pregnant females, P4 is maintained at the baseline, apart from a slight elevation in January, and E2 demonstrates a sudden hike from November and remains elevated until February, suggesting the onset of ovulation. The serum testosterone concentration in males peaked during October, which is in sync with the female ovulation period. As a result that their major reproductive events, ovulation, mating, and parturition, all transpire in November-March. Evidence also supports that Chinese pangolins exhibit signs of postimplantation (pregnancy) ranging only from 5 to 6 months (May–Oct), preceded by possible facultative delay implantation triggered by lactation. The provided data not only fill in the knowledge gap for this critically endangered species but can also assist in making informed decisions, which can directly affect the successful breeding of this species in captivity.

## Introduction

Reproductive biology is an important aspect of conservation biology; if understood adequately, the resulting benefits can be applied to a range of endangered/critically endangered fauna. However, an inevitable shortage in the reproductive biology of lesser-known species has led to the failure of several well-intentioned population recovery efforts^1^.

To date, for some critically endangered species (especially carnivores), captive breeding programs have become the face of conservation action plans. The successful conservation plans for species, i.e., black-footed ferret (*Mustela nigripes*)^2^, Iberian lynx (*Lynx pardinus*)^3^, and giant panda (*Ailuropoda melanoleuca*)^4^ retrospect how reproductive science can contribute to species conservation.

Pangolin (Family Manidae), a phylogenetic relative of carnivores^5^, tops the list since the species lacks substantial unparalleled insight into the reproductive process. There are eight identified species, i.e., four in Asia and four in Africa^6^. The Chinese pangolin (*Manis pentadactyla*) is one of the four extant species in Asia. The Taiwanese pangolin (*Manis pentadactyla pentadactyla*) is a subspecies of Chinese pangolin, endemic to the sub-tropical island of Taiwan^7^. However, the subspecies cannot be morphologically distinguished because of a lack of cytogenetic analysis conformation^8^; therefore, in this article, the Taiwanese pangolin is used to refer to the Chinese pangolin population. For Asian pangolin species (*Manis* sp.), a number of institutions, such as Taipei Zoo (Taiwan); Night Safari (Singapore); Carnivore and Pangolin Conservation Programme (Cuc Phuong National Park, Vietnam); Cat Tien National Park (Vietnam); Angkor Center for Conservation of Biodiversity (Cambodia); Conservation International and Forestry Administration (Cambodia); Indonesian Institute for Sciences (Indonesia); Research Base for Pangolin Domestication and Breeding from South China Normal University (China); Yunnan Wild Animals Park (China); and Breeding Center for Pangolin (Nandan Kannan Zoological Park, India), have been working tirelessly to achieve successful captive breeding prospects^9^. However, the birth success rate of these species in captivity is still low in general because of the lack of information on fundamental aspects of reproductive cycles^10^. Thus far, Yan, Zeng^11^ have presented the most successful records of 49 captive-born Sunda pangolins (*M. javanica*) produced from 33 wild rescued individuals. In addition to successful captive breeding, this study also documented several important reproductive phenomena based on behavioral observations^11^: (1) fertile mating can happen shortly after giving birth; (2) mating can happen in late pregnancy; (3) females may undergo the phenomena of induced ovulation. However, all these events lacked hormonal evidence.

Few captive reports share the consensus that Chinese pangolins and Sunda pangolins can undergo sexual maturity from 6 months old to 1 year old^11–14^. The weaning age in Sunda pangolins is reported to be approximately 4 months^11,14^, whereas, in Chinese pangolins, it is recorded as 5 months^15,16^. Additionally, pangolins generally hold a litter size of one pup at a time, and the pups have a low survival rate in captivity^17^, except for one male Chinese pangolin that sired two second-generation captive-bred offsprings and    survived for more than 20 years in Taipei Zoo^14,18^.

The birthing cycles in Sunda pangolins are known to occur around the year^11,14^. In contrast, 16 wild births (2010–2014) for the Chinese pangolins were observed from September through March, and among them, ten births (62.5%) were observed during December and January (K.J.-C. Pei, unpublished data). Similar birthing behavior of the Chinese pangolins was also observed in captivity (Table 1). Therefore, the present study of reproductive steroids studies is critical and would assist in substantiating the extant information on the Chinese pangolin.

**Table 1:** History of breeding, parturition, and gestation of Chinese pangolin species.

Case no.	Species	Mating date	Parturition date	Estimated gestation length	References
1	Chinese pangolin (*Manis pentadactyla*)	Between 1 and 30 Jun 1984, when the pair were housed together	3 Jan 1985	187–216 days	^28^
2		After 15 Jun 1998, when the pair were housed together	28 Nov 1998	< 166 days	^29^
3		23–27 Dec 2005, when the pair were housed together	9 Nov 2006	317–321 days	^12^
4		Before arrival on 7 Nov 2005	20 Sept 2006	> 317 days	^12^
5		Before arrival on 2 Oct 2006	9 Oct 2007	> 372 days	^12^
6		Between 8 Mar and 20 Apr 2011, when the pair were housed together	19 Oct 2011	182–225 days	^13^

Reports on gestation length for Chinese pangolins have exhibited major inconsistencies in the reproductive knowledge gathered from visual observations of their behaviors in captivity. The majority of earlier captive observations on the birth of Asian pangolins provided insufficient information on the gestation length because the individuals rescued were gravid on arrival, and babies were born unexpectedly within a short duration of time, ranging from a couple of weeks to 3 months after arrival^19–22^. So far, there have been six captive records for the Chinese pangolins, which estimate their gestation length (Table 1). These records were referable because non-pregnant females were artificially paired with males for a finite period of time before they gave birth (Table 1: Case 1–3), or females were housed alone after arrival, and births were recorded after a considerably long period of time (Table 1: Case 4 and 5).

The discrepancies in the gestation length were evident in the Chinese pangolin; not only were the longest gestation length estimations more than 12 months, but there was also an extensive difference of approximately 7 months between the shortest (< 166 days) and the longest (> 372 days) estimations. It is titillating to witness that such atypical findings in Chinese pangolins deviate from the general understanding of the average physiological (gestation) and developmental cycles in homeothermic species that are proportional to their adult body size. A long gestation period of more than 300 days is witnessed only in mammals belonging to the order Artiodactyla (giraffe, Bactrian camel)^23,24^, Proboscidea (elephants)^25^, Perissodactyla (rhinoceros)^26^, and 18 other different species such as ursids, cetaceans, and all carnivores weighing over > 100 kg^24,27^.

For the very long gestation lengths (i.e., Case 3–5 in Table 1), Zhang and Wu^13^ suggested that they resulted from environmental stress that altered females' reproductive hormones. The other plausible explanation proposed by Chin and Lien^12^ states that such discrepancies could be the result of delayed implantation (i.e., embryonic diapause), where blastocyst enters diapause and does not implant immediately; instead, it floats in the mother's reproductive tract and prolongs the gestation period^30,31^. Sun and Pei^15^ also proposed the hypothesis of delay in the Chinese pangolin. Xenarthrans (giant anteater, *Myrmecophaga tridactyla*; nine-banded armadillo, *Dasypus novemcinctus*), which share various behavioral and morphological similarities with pangolins also known to exhibit the phenomena of delay implantation^32–34^. Amid different theories, therefore, the endocrinological findings can provide substantial evidence to unravel the truth behind pangolin reproductive physiology. It is also essential because missing the reproductive endocrinological profile is one of the major setbacks for pangolins to breed successfully in captivity. Other factors such as diet, low, and compromised immune systems were also postulated to be factors that affected the success of pangolin breeding in captivity^9,12,35,36^.

It is well known that the interplay of reproductive hormones (namely, estradiol, progesterone, and testosterone) generates chronological series of events where ovulation is succeeded with mating, pregnancy, parturition, and lactation. Such endocrinological priming triggers spontaneous ovulation in various mammals where females show continuous cycling of reproductive hormones^37,38^. Cycles of this type appear to rule rodents, ungulates (except Camelidae), canid carnivores, and higher primates^39^. Sporadically, spontaneous ovulation is accompanied by a major reproductive adjustment of post-partum estrus development^40^. In some cases, post-partum estrus is integrated with delayed implantation^40^.

The present study established the annual cyclic profile of gonadal reproductive steroids, including serum estradiol (E2) and progesterone (P4) in females and testosterone (T) in male Chinese pangolins, extant in Taiwan. Apart from determining the annual reproductive endocrinological profile, this study also aimed to validate the following observations or predictions:

1. Chinese pangolin's reproductive seasonality (seasonal vs. aseasonal)^15,41–43^.
2. The ovulatory pattern in Chinese pangolins (spontaneous^15^ vs. induced ovulation^11^).
3. Chinese pangolins demonstrate potential for delayed embryo implantation or embryonic diapause^12,15,44,45^.

## Results

Among thirteen mature birth records gathered in this study, 80% of births in the zoo fell between September and December, with cases 2 and 24 giving birth in August and case 29 giving birth in March (Supplementary Table S1). Zhang, Wu^13^ also witnessed parturition between September and February, with few cases in August. The records suggest that these births were within the regular birth season of Chinese pangolin. The youngest females who gave birth in Taipei Zoo were approximately 2 years old (Case 8, 9, 15, 24, 27, and 29) (Supplementary Table S1). However, intensive monitoring examining their mating behaviors was not undertaken; therefore, no gestation period was determined for the parturition events.

**Figure 1:**
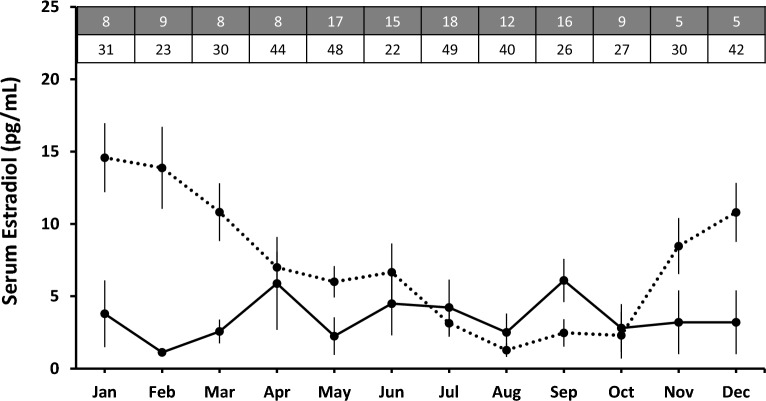
Annual serum endocrinological pattern of sex steroid E2 in pregnant and non-pregnant female pangolins. The dotted line represents the non-pregnant females, and the solid line represents the pregnant females. The grey boxes indicate the total number of samples obtained per month from non-pregnant females, and the white boxes indicate the total monthly serum samples obtained from pregnant females.

The serological endocrine findings showed that E2 (pg/mL) concentrations in pregnant females did not alter significantly annually (F_129,11_ = 0.63, p = 0.07) and asserted at/below the baseline hormonal concentration year-round (Fig. 1). On the other hand, the levels of E2 in non-pregnant females fluctuated significantly throughout the year (F_411,11_ = 7.55, p < 0.001) and demonstrated a clear seasonal pattern. In non-pregnant females, an abrupt elevation in the E2 levels was witnessed in November–December and reached its peak during January–February; the level then started to decline gradually from March and attained the lowest levels in August (Fig. 1). Non-pregnant female cycles possess a significantly higher serum E2 concentration than pregnant female cycles (F_2,358_ = 39.87, p < 0.001).

**Figure 2:**
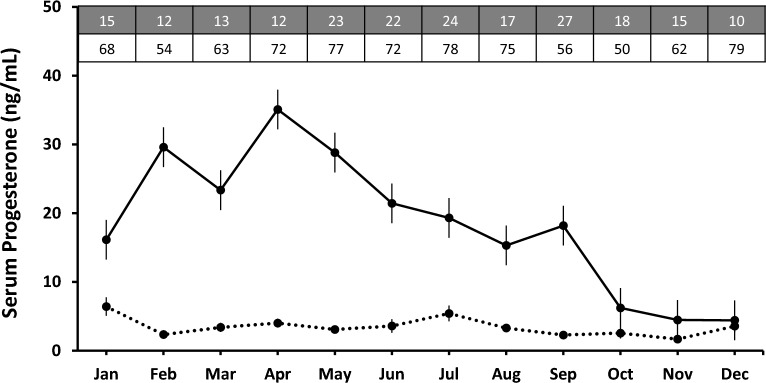
Annual serum endocrinological pattern of sex steroid P4 in pregnant and non-pregnant female pangolins. The dotted line represents the non-pregnant females, and the solid line represents the pregnant females. The grey boxes indicate the total number of samples obtained per month from non-pregnant females, and the white boxes indicate the total monthly serum samples obtained per month from pregnant females.

Opposite to the findings of E2, there was significant variation in monthly levels of serum P4 in both pregnant (F_207,11_ = 7.28, p < 0.001) and non-pregnant (F_805,11_ = 2.56, p < 0.05) females, the pregnant females demonstrated a significantly higher level of P4 values year-round than non-pregnant females (F_2,227_ = 419.98, p < 0.001) (Fig. 2).

The annual serological levels of P4 in pregnant females displayed discernible seasonal fluctuations, i.e., P4 rose quite rapidly from January (16.14 mg/mL) and attained a peak in April (35.08 mg/mL), and with a decline from May until October, it was maintained at a lower level in November–December (Fig. 2). Quite differently, the serological P4 levels in non-pregnant females were maintained at the baseline (annual average = 3.1, SE = 0.6) throughout the year, with a modest peak in January (6.4 mg/mL).

**Figure 3:**
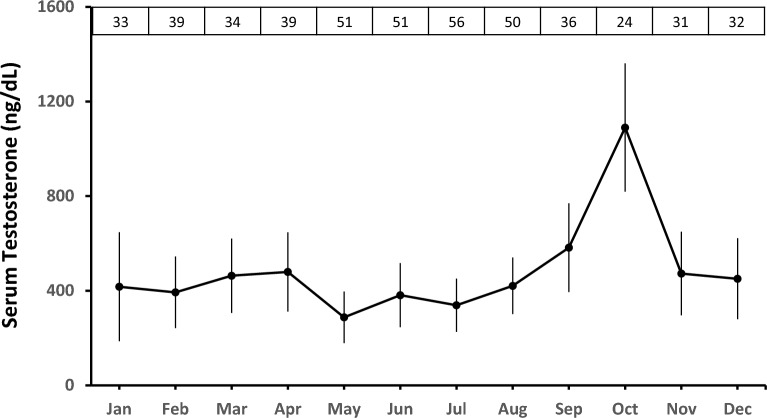
Annual serum endocrinological pattern of the sex steroid testosterone in male pangolins. The white boxes indicate the total number of monthly samples from different male pangolins.

The gonadal sex steroid concentration in males revealed that monthly T (ng/dL) levels remained low throughout the year (F_11,475_ = 1.2, p = 0.27), except for a significant peak in October (Fig. 3). During October, the serum T concentration (1089.9 ng/dL) was significantly higher than in the other months (F_2,24_ = 7.8, p < 0.05).

## Discussion

**Figure 4:**
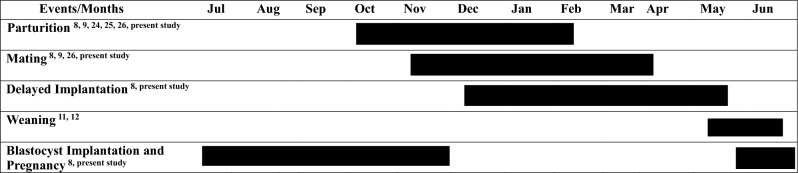
Summarization of major reproductive events in Chinese pangolin (*Manis.pentadactyla*).

The hematological analysis of reproductive steroids, i.e., E2, P4, and T, provides the novel specifics of the Chinese pangolin reproductive cycle. The present study corresponds to E2 and P4 roles in reproductive behavior in female Chinese pangolins^46^. The hormonal investigation of the annual reproductive cycle in male and female Chinese pangolins using sex steroids, and together with previous understanding^15,47^, revealed that the Chinese pangolin is a seasonally breeding species (Fig. 4), i.e., parturition ranged from August to March, but the birth peak was observed from October through December, corresponding to the field records of the Chinese pangolin^15,16^ (K.J.-C. Pei, unpublished data). The mating event transpiring from November through March is accompanied by a prolonged gestation period observed from January to September. Similarly, serum T concentrations elicit that male Chinese pangolins undergo sperm production to match the reproductive cyclicity (Nov-Jan) in females by exhibiting peak testosterone levels during October. Thus, a significant rise in serum T levels overlaps with the event of parturition in females, thus generating a sync in both sexes' reproductive behavior.

The present hematological study of reproductive steroids of the Chinese pangolin has also been backed up by substantial field observations/reports and variate from the captive findings of the Sunda pangolin^11^, suggesting that the reproductive biology of these two species is different. The findings in the case of the Sunda pangolin might require further field evidence(s) to corroborate with captive reports.

### Reproductive physiology  of Chinese pangolin

In mammals, females marked the estrus behavior by rising estradiol-17β (E2) levels in the serum^46^. The significant seasonal pattern in serum E2 in non-pregnant pangolin females suggests that the Chinese pangolin exhibits ovulation that begins either at or immediately after the heightened period of E2 during November- March (Fig. 1). The hematological rise in the serum E2 in Chinese pangolin coincided with the behavioral estrus observations in wild Chinese pangolin, for which their copulatory occasions were reported from November-March^15^. This E2 observed is likely of ovarian origin, demonstrating that Chinese pangolins ovulate spontaneously during the same period. Pangolins are the mitogenomic mirror of Carnivora, and such idiosyncrasy of spontaneous ovulation is clearly witnessed in various caniform species such as wolves, jackals, coyotes, and canines^46,48–50^. Species like Asian small-clawed otters (*Aonyx cinereus*), North American river otters (*Lontra canadensis*), American mink (*Neovison vison*), stoat (*Mustela erminea*), and giant otters (*Pteronura brasiliensis*) also demonstrate spontaneous ovulation^51^.

Whereas the circulating P4 in non-pregnant females was maintained at the baseline (Fig. 2) as published in Chin, Lien^12^. However, the circulating levels of P4 in non-pregnant females began to rise in December and hiked during January (Fig. 2), after a concomitant rise in the serum E2 levels, i.e., November (Fig. 1), suggesting that P4 also plays a critical role during estrus behavior. Similarly, in highly seasonal canid species such as African wild dogs (*Lycaon pictus*), the female reproductive cycle's estrus phase is characterized by elevated E2 and P4 levels^52–56^.

The abrupt rise in P4 values, accompanied by the elevated E2 levels, suggests that the circulating P4 is of follicular origin^57^. Studies on armadillos demonstrated that ovulation occurs spontaneously in unmated individuals, which leads to the formation of the corpus luteum (CL), which is grossly and histologically indistinguishable from the CL formed during pregnancy^58^. The present study indicates that the formation of CL from December onward could be responsible for heightened levels of P4 in the serum.

From March onwards, the levels of serum E2 exhibited a declining pattern in non-pregnant females, ensembling the termination of estrus behavior in females. The hyper-elevation of E2 for a brief period during November–March indicates that the species are clearly seasonal breeders. The remaining months are attributed to the anestrus behavior, likely to be caused by the stimulatory effect of the decrease in the E2 to P4 ratio, a mechanism responsible for the abrupt termination of estrus behavior in female mammals^57^.

The phase of pregnancy in females was marked by non-significant dissimilar annual E2 concentrations in the serum. Contrastingly, the circulating P4 levels fluctuated significantly annually with prominent sustained levels until parturition. Continuous production of P4 is required to develop and maintain pregnancy^46^. The P4 orchestrates various responsibilities, including the differentiation of the endometrium, suppressing uterine contractability, and maintaining its integrity and the attachment of the placenta^59^. Elevated P4 hormone levels in pregnant females are likely associated with P4 sex steroid production from the CL. In many canid species such as red wolf (*Canis rufus*), maned wolf (*Chrysocyon brachyurus*), and domestic carnivores, the production of pregnancy-associated P4 is produced by the CL and is significantly higher than in non-pregnant females^52,59,60^ as witnessed in our studies. However, the observed levels of E2 hormone align with domestic carnivores^61,62^.

In species such as rabbits and various carnivores, the secretion of E2 is essential for sustaining the functionality of the CL^63–66^. Studies conducted on rabbits and rats revealed that E2 is secreted by luteal cells and plays a role in luteal regulation^67–70^. E2 forms an association with prolactin (PRL) in species like pigs to sustain the luteal function and stimulate P4 secretion^71^. E2 assists along with the luteotrophic hormone, PRL, in many other carnivorous species to regulate the function of the CL to assist pregnancy^70,72^. Thus, a gradual rise in circulating E2 towards the end of the year possibly triggers post-partum estrus behavior in pregnant females.

However, the recurrent estrus cycle is a rare mechanism in mammals as females are believed to spend their entire lives being in seasonal diestrus, pregnant, or lactating^40,73^. Thus, the Carnivora, especially pinnipeds and Rodentia with short reproductive life cycle post-partum estrus, enables the female to fit pregnancies in the breeding season concurrently with lactation^74–76^. Therefore, post-partum estrus behavior in pangolins enhances the chance to conceive and leaves a substantial number of progeny as witnessed in rodents bearing a large number of pregnancies that are known or presumed to occur from post-partum mating^77,78^.

Pregnant pangolin females showed an abrupt decline in serum P4 during October–December is a typical of the birthing event in Chinese pangolins. Characteristically, a significant decline in levels of P4 will occur before parturition in response to the maturation of the fetal pituitary-adrenal axis and in the elevation of fetal glucocorticoids^79^. The receding levels of P4 also facilitate other endocrine and paracrine factors responsible for uterine contractibility, cervix softening, and relaxation of the birth canal^80–82^. Thus, the decline in the circulating serum levels of P4 during October–December in pregnant females is consistent with the previous direct field and captive observation records that pangolins are born between December and January^12,13,16,20,22,83^. Physiologically, the CL regresses and ceases its function of production of P4 after parturition; however, in species such as Eurasian lynx (*Lynx lynx*)^84^, Iberian lynx, and Canadian lynx (*Lynx canadensis*)^85^, the complete luteolysis does not occur and causing the circulating levels of serum P4 remains relatively elevated after the parturition, turning lynx into a monoestrous species. Therefore, it is highly likely that elevated P4 levels in Chinese pangolins from January through September (~ 9 months) as the presence of P4 after parturition may impart to species adaptability to environmental stressors to ensure the birth of only one litter per year^86^. Therefore, the Chinese pangolin experiencing nearly similar nature of P4 hormones suggests monoestrous behavior in the respective species.

Comprehensively, the Chinese pangolin shares similarities with highly seasonal canid species, i.e., the female reproductive cycle's estrus phase is characterized by elevated E2 and P4 levels^52–56^ followed by reproductive synchrony with males.

Male Chinese pangolins showed abrupt seasonality with the highest serum T concentrations that only occurred in October (Fig. 3). The heightened serum T concentrations aligns with the parturition records in females. As mentioned earlier, parturition likely triggers post-partum estrus accompanied by ovulation in female Chinese pangolins. Hence, the copulation behavior initiated by high serum T concentrations in males at this stage enhances the chances of conception. It is observed in carnivores with a shorter breeding window, such as in spontaneous ovulators, that the males tend to have higher sperm production to keep up with the female estrous synchrony^87–91^ and provide males with the chance to sire more offspring's^92^. Due to the solitary lifestyle, pangolin sexes interact for a short period, so this may cluster all the reproductive events into a short time interval. Hence, the reproductive behavior of the male Chinese pangolins presented a high degree of seasonality in sperm production. Conclusively, the major reproductive parameters, including ovulation, mating, and parturition, all transpire from November to March.

This evidence demonstrates that Chinese pangolins are seasonal breeders and ovulate spontaneously, i.e., continuous seasonal breeding cycle. This hematological evidence of reproductive steroids suggests that the Chinese pangolin species can behave differently physiologically from the Sunda pangolins, exhibiting aseasonal reproductive behavior and induced ovulation^14^. A pronounced elevation in serum P4 from January to September was reflected in our study, which suggested that the Chinese pangolin has a long gestation period, as reported in the introduction. Moreover, the variation in the gestation period (~ 9 months) is likely to be associated with delayed implantation of the embryo, which could explain the unusual prolonged pattern of progesterone hormone in such small carnivores.

### Evidence for delayed implantation

The hormonal pattern of E2, P4, and T showed a synchronous pattern to maximize the output into a successful copulation event from November through March. The current study has shown an anomalous reproductive pattern of discernibly heightened P4 for a continuous period of ~ 275 days ranging from January to September in pregnant females. However, such observations are astonishingly contrasting from the small mammals' records belonging to Canidae, Procyonidae, Mustelidae, Viverridae, Hyaenaidae, and Felidae, weighing approximately 6 kg. They have gestation lengths of 55, 80, 64, 65, 100, and 67 days, respectively^93^, corresponding to heightened levels of P4 for a very brief period of time. Such distinct anomalous behavior of elevated gonadal steroid P4 indicates that Chinese pangolin may exhibit the phenomena of delayed implantation or embryonic diapause.

The species, such as roe deer (*Capreolus capreolus*)^94^ and nine-banded armadillo^58,95^, revealed a high mean concentration of P4 in both pregnant groups and the females exhibiting delay/diapause without being significantly distinct from each other. Therefore, suggesting a possibly similar role of P4 in pangolins as seen in some carnivores.

Moreover, a serological analysis report of the rehabilitated Chinese pangolin during 2012–2018 by Pingtung Rescue Center for Endangered Wild Animals (PTRC), Taiwan, showed elevated pituitary hormone, prolactin from December to April, followed by elevated gonadotropins E2 and P4^47^. The elevated luteotrophic hormone PRL suggests its role in ovulatory mechanisms and mating^47^. The Chinese pangolin also exhibits the maternal instinct of lactation (nursing) from December to May^15,16^. Because of nourishing the newborn pup and undertaking the successful mating event during the brief interaction. The short interruption caused by lactational diapause helps the mother invest and mobilize more nutrients in the maternal care of the pup because lactation is often more costly than pregnancy, i.e., it causes a substantial drain of the nutrients^96^. Such intensive mobilization of nutrients enhances the chances of survival of the born pup over the unborn individual^97^. Thus, the sequential release of the offspring can assist the mother in committing nutritional resources to rear the conceptus and undergo a new term of pregnancy. The field observations strengthened the endocrinological findings, where the nursing period (lactation and weaning) ranged from 22 to 23 weeks (beginning from early December to early May)^16^. De Lange^98^ performed an anatomical examination on pregnant females and observed a free-floating blastocyst in the uterine lumen. In comparison, Sweeney^99^ observed a well-developed fetus in the Temminck pangolin (Smutsia temminckii) during the nursing period suggesting a different reproductive behavior from the Chinese pangolin; however, needs more studies for further validation.

Additionally, in early August, the ultrasonographic scanning carried out on a rescued wild Chinese pangolin by PTRC showed the fetus's presence in the uterus. Sun and Pei^15^ showed the fetus's crown lump length (CRL) was 25 mm, witnessed after approximately 3 months of maternal separation. The combination of endocrinological, field, and ultrasonic findings provided shreds of evidence that despite sustained elevation in P4 (January–September) for 9 months, pangolin exhibits a phase of delay before implantation. Therefore, the Chinese pangolin exhibits signs of postimplantation (pregnancy) ranging only from 5 to 6 months (May–October), preceded by a delay that is likely to be caused by the nursing behavior of the individuals, suggesting that females undergo facultative embryonic diapause triggered by physiological phenomena of lactation^100,101^.

Furthermore, our studies showed that the pregnant females experienced a mild hematological shift in the mean concentration of P4 (28.8–6.2 mg/mL) from May to October. The hematological difference suggests that the Chinese pangolin is highly likely to exhibit diapause, possibly occurring from January to May (~ 5 months). The postimplantation period may be estimated to be 5–6 months, from May–October. Because of possible metabolic extraction of P4 from plasma by the gravid uterus for implantation, as witnessed in many domestic carnivores^102,103^.

Sun and Chang^83^ established that the Chinese pangolin demonstrates an acute polygynous mating system; therefore, exhibiting the phenomena of diapause may provide many opportunities for the females to perform elaborate maternal care of the young ones, as well as, the sexual selection may provide greater benefits to females to choose to copulate with the fit but polygynous male^104^. Boyce^105^ showed that mammalian species showing polygynous mating systems possess high maternal investment at the birth of the offspring; therefore, the Chinese pangolin showing the mechanism of delaying the new pregnancy possibly ensures the survival and the maturation of the existing offspring. Apart from maternal care, delayed implantation permits the mammals to mate during the most favorable time of the year^106^. Bearing diapause, especially in small solitary carnivores, provides a longer time for the pup to establish territories and perfect its hunting skills^106^ to combat unfavorable conditions. It is quite possible that embryonic diapause in Chinese pangolin occurs, possibly due to lactation providing a greater adaptive advantage to the newborn pup to attain nutrition and skills prior to exhibiting territorial behavior.

Our study provided evidence of delayed implantation in Chinese pangolins, which coincides with the hypothesis.

### Conservation breeding and colony management implications

This study's majority of parturition occurred between September and December, coinciding with the abrupt decline in serum P4 concentrations (Fig. 2). It was also consistent with the field observations (K.J.-C. Pei, unpublished data)^15^ and captive records^13^. Physical field observations validated the reproductive cycle of pregnant females with the help of the hematological monitoring of reproductive steroids demonstrated in this study.

Interestingly, despite being physiologically feasible, as shown by the current study's hormonal profiles and cumulative records from the wild^15,16^ no consecutive births were given in our study. This was because the present study's pairing was paused during the parturition season so that the male would not interfere with the mother, which accidentally also forbids copulation. Therefore, captive management in the future might be adjusted if increasing productivity is the goal.

The current studies have tremendously facilitated the understanding of this species's biology. For the management of critically endangered species (or sub-species), such as the pangolin, captive breeding might be considered an essential part of conservation strategies^107^. The ex-situ population acts as a genetic repository stock to strengthen the numbers in the wild population counterparts^3,107–109^. So far, the only black-footed ferret, Iberian lynx, and giant panda have exploited reproductive sciences for conservation programs^2,3^. To successfully breed individuals via conservation breeding programs, the selection of reproductive pairs based on their genetic importance in the extant population status is critical^110^. Therefore, the diagnosis of early pregnancy would be of great interest. The present study's serum diagnosis of gonadal hormones definitely provided more accuracy to the existing reproductive information.

Like other carnivores, breeding management of solitary Chinese pangolin requires reliable estrus detection to execute pairing with males when the female is in heat^110^. The gonadal steroid P4 can be a useful marker of pregnancy in Chinese pangolins because pregnant females illustrated prolonged high levels past the breeding phase, opposite to non-pregnant females. The establishment of a hormonal reproductive profile to contemplate the reproductive events- ovulation, copulation, and pregnancy-could also assist the scientist in building valuable reproductive strategies and technologies that will greatly benefit the captive management of pangolins.

To the authors ' knowledge, the study is the first in cumulative annual account of estradiol, progesterone, and testosterone in the pangolins. This study provides additional information on understanding the phenomena of potential delayed implantation in Chinese pangolins. However, many questions still remain, and many studies, such as understanding the role of LH, FSH, and PRL in pregnant and non-pregnant females, still warrant further investigation. The dearth of histological analysis to develop an in-depth understanding of the functionality and the presence of the CL, at various stages, including pre-implantation, implantation, and postimplantation, wanting more focus and can be further helpful to augment the findings of the present study. Vaginal cytology, i.e., assessment of vaginal cornified cells, along with vulval dimensions, can be informative and a practical tool along with the endocrinological detection of estrus and various reproductive stages in pangolins. Answers to these questions should allow us to successfully advance in devising breeding plans to enhance captive breeding in these reclusive and endangered mammals.

## Materials and methods

### Ethics statement

The periodic physical examination and hematological sampling were performed under the guidelines of the Taiwan Forestry Bureau (permit number 1071700430) as required by the Wildlife Conservation Act, 2013. Ethics approval for our animal use protocol for blood withdrawal was granted by the Institutional Animal Care and Use Committee, Taipei Zoo (permit number 10602). Well-trained and experienced veterinarians carried out all clinical monitoring and examinations following the protocol prescribed in Chin, Lien^12,111^. The methodology used in the study complies with the ARRIVE guidelines and regulations.

A total of 34 females and 29 male pangolins rehabilitated at Taipei Zoo during 2004–2017 were employed for this hormonal study. The bodyweight of rehabilitated pangolins, except 3 males and 3 females, was measured upon arrival. The heaviest weight was more than 5 kg in females, and more than 7 kg in males, and the lightest weighed approximately 100 g, which were newborns (Supplementary Tables S1, S2). Based on their body weight, the estimated age on arrival was attributed based on long-term field monitoring of 19 females and 16 males after birth (K.J.-C. Pei, unpublished data): both sexes are 1 kg at 6 months old, 2 kg at 1-year old, and females reach 3 kg at 2 years of age, and 4 kg at 3-years old, while males reach 4 kg at 2-years and 5 kg at 3-years of age, respectively.

The individuals were isolated and housed in individual pens after arrival as per the protocol of Chin, Lien^12^. They were fed a cooked meal every day at 4 PM^12,29^, and ad libitum water. Individuals of both genders were not cohabitated systematically. Approaching behavior toward the opposite sex was the catalyst for the cohabitation process. Females were housed individually once mating behavior was observed until they gave birth. For those females who gave birth, no pairing was attempted for several months after giving birth. Periodical physical examination and serum sampling by trained veterinarians was performed for these individuals. During the physical examination, the pangolins were anesthetized by inhaling an anesthesia mixture of 5% isoflurane and oxygen with a flow rate of 6 L/min through a gas chamber. Pangolins were removed from the gas chamber once they started losing their response. Clinical variables such as heart rate, blood oxygen saturation (SpO_2_), and rectal temperature were carefully monitored^111^. The blood samples were withdrawn using a 1.25 inches needle carefully inserted to the depth of 2 cm in the coccygeal vein along the tail's ventral midline. Blood samples were kept at room temperature for 6–7 h until the blood clotted, and serum was separated after centrifugation for 10 min at 2000 rpm. The serum samples were stored at − 20 °C until analysis.

### Serum collection

All the pangolins were sexually mature, i.e., bodyweight (> 1.5 kg)^14,112,113^ at the time of serum procurement for hormonal analysis. Among 34 females, 10 females gave a total of 16 births in Taipei Zoo (Supplementary Table S1). Among those 16 births, eight females (Cases, i.e., 2, 8–9, 21, 24, 26–27, and 29) gave 13 births to mature cubs weighing between 97 and 140 g. Females 10 and 15 gave birth to immature cubs weighing 52 g and 80 g, respectively, and two females, i.e., 9 and 29, delivered cubs more than once during the period of the study. All these cubs died within a duration ranging from days to months (Supplementary Table S1). Additionally, four females (i.e., 1, 12, 21, and 19) were gravid on arrival and were diagnosed with immature fetuses confirmed with autopsy records after mortality (Supplementary Table S1).

In order to compare serum hormone profiles between females with different reproductive statuses, all-female serum samples were segregated into pregnant and non-pregnant samples. Serum samples obtained 12 months before parturition were pooled to represent the pregnancy samples. The non-pregnant serum samples were those sets of samples collected from females who did not exhibit any signs of parturition/pregnancy during the study period or pregnant females after parturition until 12 months before the next parturition. This kind of sampling design is inevitable for sensitive, difficult to keep alive in captivity, and less known species like a pangolin.

Among the 29 male individuals, four were born in Taipei Zoo (cases, i.e., 21, 23, 27, and 29); case 23 is extant in captivity (Supplementary Table S2).

### Serum analysis

The serological samples obtained were processed and analyzed for progesterone (P4), estradiol-17β (E2), and testosterone (T) using radioimmunoassay (RIA) at RIA lab, Lezen Reference Lab, formerly known as Chiu Clinic (3rd Floor, No.2, Nan-king West Road, Taipei, 104, Taiwan). The hormonal assays were performed using the available commercial kits (Beckman Coulter^R^) for quantitative measurements of sex steroids (P4, E2, and T) previously validated in several carnivores and bovines^114–116^. All analyses were performed in duplicates.

### Progesterone radioimmunoassay

The concentration of serum P4 was determined in duplicates using the commercial double-antibody DSL ^125 ^[I]Progesterone RIA Kit (DSL-3400; Beckman Coulter Inc., Brea, CA, USA). Intra and inter-coefficient of variations (CV%) were 5.4% and 3.8%, respectively. The smallest detectable dose was 0.1 ng/mL, the steroid recovery was > 95%, and the recovery linearity was > 91%. This assay has the following significant cross-reactivities: 100% Progesterone, 2.36% 17α-hydroxyprogesterone, 0.36% Pregnenolone, 0.08% Cortisol, 6.08 Corticosterone, 0.66% 20α-dihydroprogesterone, 1.38% Medroxyprogesterone.

### Estradiol-17β radioimmunoassay

Serum E2 was assessed using the commercial RIA kit DSL ^125^ [I] Estradiol-17β (DSL-4400; Beckman Coulter Inc., Brea, CA, USA). This assay's cross-reactivities were as follows: 100% Estradiol, 0.01% Estrone Sulphate, 1.98% Estrone, 0.50% Estriol, 0.002% Estriol 17-sulphate, 0.37% Ethinyl estradiol, 0.29% Estradiol valerate, 0.04% Testosterone, 0.001% Androstenediol, 0.35% 17α-estradiol, and 0.029% 17β-estradiol-glucuronide. The intra-assay precision (CV%) was 5.6%, and the inter-assay precision (CV%) was 3.53%, with the lowest detectable concentration with a 95% confidence interval was 2.7 pg/mL. The spiked recovery of E2 was 104%, and the mean recovery linearity was 110%.

### Testosterone radioimmunoassay

We used RIA kit DSL ^125^ [I] Testosterone (DSL-4100; Beckman Coulter Inc., Brea, CA, USA) for serum T analysis. The assay sensitivity with 95% confidence was 0.01 ng/mL. The recovery of added steroids determined by spiking yielded a mean recovery of > 95%, and recovery linearity was > 91%. The cross-reactivities of T with other steroids were: 100% Testosterone, 0.4% Testosterone-glucuronide, 0.3% Testosterone-sulfate, 2.0% 5-alpha-DHT, 0.4% Androstanediol, 0.6% Androstenediol, 0.7% Androstenedione, 0.2% Androsterone, 0.2% Estriol, 0.4% Estrone, 0.4% Progesterone, and 0.5% 19-Hydroxytestosterone. The intra and inter-assay coefficient of variations were 2.71% and 5.65%, respectively.

### Statistics and reproducibility

Calculations of serum concentration baseline values were performed using an iterative process excluding all the values greater than the mean + 2SD^117,118^. The peaks were defined with the hormonal concentration exceeding the baseline of + 2SD. Serum P4 (N = 1014; pregnant = 208 and non-pregnant = 806) and E2 (N = 542; pregnant = 130 and non-pregnant = 412) levels of pregnant or non-pregnant females and serum T (N = 476) concentrations from different males were pooled independently to produce monthly means. The Welch Independent t-test was performed to determine differences between groups. Hormone concentrations are expressed as the mean ± SEM. The comparison of hormone concentrations among different months was made using the general linear model (GLE), and univariate analysis (ANOVA). Statistical analyses were performed using IBM SPSS (Chicago, Illinois, USA) and GraphPad Prism (SD, CA, USA) software. Statistical significance was tested to 95% level (p < 0.05), and all the tests were two-tailed. The sampling bias did occur in the study, but the bias did not affect our findings and the interpretation because we used mean values (each with enough samples) to represent monthly changes.

### Supplementary Information

Supplementary Tables.

## Data Availability

The data findings of this study are included in the manuscript and provided as supplementary files. For additional information, please contact the corresponding author.
