# Supplementary material for: New horizons in the reproductive biology of Chinese pangolin (Manis pentadactyla) using the gonadal hormonal profile
Source: Sci Rep. 2023 Oct 3;13:16630. doi: 10.1038/s41598-023-43237-0 (PMC10547839; doi:10.1038/s41598-023-43237-0)
Supplement: Supplementary file 1 — Supplementary Tables. [file 41598_2023_43237_MOESM1_ESM.pdf]

S. no	Animal ID	Date of arrival	BW(Kg) (Arrival)	Date of first collection	Date of last collection	Total samples obtained		Interval between the blood collection (days)	Date of release	BW (Kg) (Rele -ase)	Condi-tion upon release	Reprod uctive Status	No. and Date of birth of cubs	BW (g) of cubs/fetus at birth
						E2	P4							
1	Quarantine	2004/4/21	3.6	2004/5/17	2004/6/17	0	2	28	2004/10/13	2.7	D	PA	-	41
2	Little	2004/6/1	1.8	2004/6/14	2014/6/7	91	160	7-144	2014/6/8	3.38	D	P	1 (2013/8/23)	97
3	Mary	2004/10/23	3.2	2004/10/26	2005/11/4	21	21	6-22	2005/4/17	2.95	D	NP	-	-
4	Golf	2004/8/13	2.2	2004/11/10	2005/10/7	45	45	3-15	2005/10/19	1.54	D	NP	-	-
5	No7	1996/5/12	-	2005/4/4	2007/7/2	19	19	4-117	2008/2/13	3.14	D	NP	-	-
6	6D06	1998/6/15	-	2005/4/4	2005/10/3	11	10	7-41	2005/10/5	3.6	D	NP	-	-
7	Alice	2005/4/10	3.8	2005/4/4	2005/5/2	3	3	7	2005/5/7	3	D	NP	-	-
8	Sanshia	2005/7/9	2.28	2005/7/9	2015/9/24	78	142	7-192	2015/10/26	5.71	D	P	1 (2006/11/9)	110
9	Sindian 2	2005/9/19	1.96	2005/9/20	2016/11/29	70	116	7-355	2016/12/15	5.2	S	P	4 (2010/10/12; 2012/11/14; 2014/9/30; 2017/10/30)	110; 132; 133; 108
10	C1158	2005/12/8	2.8	2006/1/16	2006/10/13	37	37	4-16	2006/11/3	1.6	D	P	1 (2006/9/20)	52
11	Yuanshan	2006/5/16	1.4	2006/5/17	2006/6/5	3	3	7-13	2006/6/4	1.095	D	NP	-	-
12	C1172	2006/5/17	4	2006/5/17	2006/10/2	21	21	5-11	2006/11/2	2.8	D	PA	-	55
13	Datung	2006/5/22	2.34	2006/5/22	2012/7/2	36	76	3-169	2012/10/9	4.9	S	NP	-	-
14	New Antai	2006/5/26	2.1	2006/5/29	2006/7/31	10	10	7-9	2006/8/5	1.3	D	NP	-	-
15	Waishuangshi	2006/10/2	2.14	2007/2/1	2012/5/4	37	62	13-169	2012/7/5	3.05	D	P	1 (2007/10/9)	80
16	Yangmingshan	2007/12/31	4.06	2007/12/31	-	1	1	-	2008/1/30	3.6	D	NP	-	-
17	Quaishan	2008/6/10	1.76	2009/2/12	2009/8/31	1	9	13-104	2009/8/31	4.53	S	NP	-	-
18	Fushaingchiang	2008/8/13	1.38	2009/2/12	2015/8/31	3	36	14-247	2015/11/8	1.86	D	NP	-	-

19	Jianshan	2008/12/1	2.02	2009/5/26	2009/9/21	1	9	10-23	2009/9/24	2	D	PA	-	72
20	Jianshani	2009/5/27	4.18	2009/6/13	2011/11/21	0	13	20-348	2012/1/2	2.88	D	NP	-	-
21	Shihding	2009/8/3	5.6	2009/8/3	2015/9/2	3	50	2-169	2015/10/14	4.27	D	PA	1 (2009/12/8)	110
22	Yellowstone	2008/6/7	2.32	2009/12/13	2010/6/8	3	3	4-171	2010/7/24	3.734	D	NP	-	-
23	990526	2010/5/26	2.6	2010/5/26	-	1	1	-	2010/7/4	-	S	NP	-	-
24	Chuanbei	2010/1/3	0.11	2011/1/20	2016/10/3	0	22	27-247	2014/10/22	4.81	D	P	1 (2012/8/22)	110
25	1000406	2011/4/6	3.32	2011/4/6	-	1	1	-	2011/4/10	-	S	NP	-	-
26	20111116	2011/11/16	5.12	2011/11/21	2012/11/5	0	5	5-328	2012/12/22	3.1	D	P	1 (2012/12/13)	115
27	Chenyouwei	2011/11/17	2.3	2012/1/2	2014/4/25	0	29	27-97	2014/10/13	6.3	D	P	1 (2012/10/17)	116
28	1010707	2012/7/7	1.7	2012/11/5	2016/2/24	0	11	35-309	2018/6/27	4.68	S	NP	-	-
29	Cream	2012/8/22	0.11	2013/4/1	2016/12/28	0	36	24-79	2018/6/27	4.66	S	P	3 (2014/11/16; 2016/3/13; 2017/9/2)	115; 132; 140
30	1020314	2013/3/14	5.3	2013/4/1	-	1	1	-	2013/4/13	-	D	NP	-	-
31	1011201	2012/12/1	2.65	2013/5/6	2016/12/28	31	41	23-67	2018/6/27	4.3	S	NP	-	-
32	1020130	2013/1/30	3.9	2013/5/25	2013/6/4	0	2	10	2013/7/1	3.1	D	NP	-	-
33	1030930	2014/9/30	0.133	2015/4/30	2017/1/22	14	17	3-75	2018/6/27	5.79	S	NP	-	-
34	1040829	-	-	2015/11/2	-	1	0	-	-	-	-	NP	-	-

Supplementary Table1: Summary of female Taiwanese pangolin used for the collection of serum samples. D= Dead, S=Survived and released

Supplementary Table 2: Summary of male Taiwanese pangolin used for the collection of serum samples. D=Dead, S=Survived and released

**S.no**	**Animal ID**	**Date of arrival**	**Body weight (Kg) (Arrival)**	**Date of first Sample collection**	**Date of last sample collection**	**Total samples obtained**	**Range of intervals between blood collection (Days)**	**Date of release**	**Body weight (Kg) (Release)**	**Condition upon release**
1	Stump Tail	-	-	2004/5/19	2004/7/15	4	5-39	2004/7/16	3.5	D
2	Radio	2004/2/19	5.5	2004/5/19	2004/11/22	14	2-68	2004/12/3	4.7	D
3	New Comer	2004/5/1	5.9	2004/5/19	2005/3/21	26	2-50	2005/3/31	3.1	D
4	c1092	2003/9/27	2.5	2004/6/23	2004/7/5	3	5-7	2004/7/10	2.81	D
5	Audi	2004/7/22	1.5	2004/7/2 2	2006/9/25	84	6-68	2013/9/14	2.8	D
6	Nike	2004/11/26	3	2004/11/30	2005/4/18	14	7-14	2005/4/23	3.5	D
7	Michall	2005/3/21	5.2	2005/4/11	-	1	-	2005/4/17	4	D
8	Lucky	2005/5/30	4.6	2005/6/13	2005/6/20	2	7	2005/6/22	4.6	D
9	Sindain 1	2005/6/6	2.3	2005/6/13	2006/8/14	49	4-22	2007/3/1	2.2	D

10	C1156	2005/11/21	2.52	2005/12/5	2006/9/25	32	7-18	2007/4/5	2.84	D
11	Pingshi	2006/1/1	5.6	2006/1/2	2006/2/20	6	6-15	2006/2/20	4.2	D
12	C1161	2006/1/5	6.8	2006/1/5	2006/6/5	15	4-21	2006/6/11	4.3	D
13	C1162	2006/1/17	5.2	2006/1/22	2006/6/12	17	7-16	2006/6/13	3.4	D
14	Dungshan	2006/3/20	6.2	2006/4/3	2006/7/24	14	5-7	2006/8/11	4.5	D
15	Yangmingshan	2006/4/14	2.5	2006/4/14	-	1	0	2006/4/17	2.5	D
16	Hualien	2006/4/17	6.02	2006/4/22	2006/4/27	4	2-3	2006/4/27	6	D
17	Ludong	-	-	2006/5/13		1	0			-
18	Airport	2006/6/15	5.1	2006/6/15	2006/9/25	12	7-14	2007/12/19	3.26	D
19	Toufen	2006/6/16	2.8	2006/6/16	2006/9/25	12	7-24	2012/10/4	5.67	S
20	Chichi	2006/11/23	7.3	2014/6/28	2014/9/25	3	27-30	2014/11/22	7	S
21	MSG	2013/8/3	0.145	2014/7/28	2016/11/22	26	24-123	2016/12/23	6.46	D
22	D1600	2013/8/23	2.2	2014/7/28	2016/12/28	21	26-123	2018/6/27	4.12	S
23	13#	1997/12/11	-	2014/8/3	2016/12/28	26	24-120	2018/6/27	6.57	S
24	Shueili	2008/10/16	4.23	2014/8/25	2016/11/29	23	26-147	2016/11/29	5.74	S
25	1010702	2012/7/2	1.56	2014/8/25	2017/1/22	26	23-120	2018/6/27	4.5	S
26	1030613	2014/6/5	224	2015/2/25	2017/1/22	24	24-35	2018/5/27	5.08	S
27	1030930	2014/11/16	0.115	2015/3/31	2016/7/27	14	24-88	2016/7/16	4.1	D
28	1050225	2016/2/25	-	2016/2/29	-	1	0	2016/2/29	-	S

29	20160313	2016/3/13	0.132	2016/12/28	-	1	0	2017/5/9	1.88	D
